# The data on molecular docking of cinnamic acid amide on dengue viral target NS2B/NS3

**DOI:** 10.1016/j.dib.2022.108036

**Published:** 2022-03-08

**Authors:** Nadia Mohamed Yusoff, Asnuzilawati Asari, Siti Nor Khadijah Addis

**Affiliations:** a Faculty of Science and Marine Environment, Universiti Malaysia Terengganu, 21030 Kuala Nerus, Terengganu, Malaysia; b Advanced Nano Materials (ANOMA) Research Group, Faculty of Science and Marine Environment, Universiti Malaysia Terengganu, 21030 Kuala Nerus, Terengganu, Malaysia

**Keywords:** *In-silico*, Dengue, Autodock, Cinnamic amide, NS2B/NS3

## Abstract

A natural occurring class compound, cinnamic acid is composed of a benzene ring, an alkene double bond and an acrylic acid functional group. Due to the feasibility of its structure modifications with a variety of compounds, cinnamic acids have been actively explored to improve their biological efficacy. Cinnamic acid derivatives have been reported to exhibit an antimicrobial property. Despite the beneficial properties of cinnamic acid derivatives, the antiviral activity of the amide derivatives especially against the dengue virus is poorly defined. Herein, the cinnamic amide derivatives were evaluated for their potential as an anti-dengue virus through the *in-silico* analysis of the derivatives. This data aimed to analyze the interactions of the derivatives against the non-structural protein of viral target, dengue virus type 2 (DENV-2) NS2B/N3. The evaluation was based on binding affinity, interaction type (bond type and distance) and interaction with amino acids. Three derivatives (CAA15, CAA16 and CAA17) with the best docking score were reported. Enhanced understanding of the interaction acquired from this analysis provide a useful information on for the prediction of the binding behavior affinity of cinnamic amide derivatives and is ultimately useful in the rational design of drugs to synthesis new compounds with the potential benefits against DENV-2.

## Specifications Table

**Table:** 

Subject	Chemistry
Specific subject area	Molecular docking
Type of data	Table Image Figure
How data were acquired	Molecular docking (AutoDock 4.2), ChemDraw Professional 16.0, OpenBabel GUI, Discovery Studio 2020 Client.
Data format	Raw Analyzed
Parameters for data collection	Docking score and interaction of the ligand with amino acid residues in the binding pocket
Description of data collection	The structure of cinnamic amide derivatives was constructed and energy minimized using ChemDraw software. The minimized structures were docked on selected anti-viral targets using AutoDock software.
Data source location	Institution: Universiti Malaysia Terengganu City/Town/Region: Kuala Nerus, Kuala Terengganu Country: Terengganu
Data accessibility	Tables and Figures of the docking are accessible in the article. Molecular docking files for Figure 2–4 are available at https://data.mendeley.com/datasets/838gm89sr2/1. doi: 10.17632/838gm89sr2.1

## Value of the Data

• The data provide on the interaction between cinnamic amide derivatives with the viral target NS2B/NS3 protease (NS2B/NS3pro) protein.
• The *in-silico* analysis of the antiviral properties of cinnamic amide derivatives may indicate the direction for future research in the field of anti-dengue therapy.
• The screening data help minimized research time considerably by enable the researchers to rapidly identify promising compounds and its interaction with the viral target.
• The data are useful for research scholars with insufficient software and hardware requirements which not affordable by them.
• The data can facilitate on the direction of the functional design of cinnamic amide derivatives specifically to target dengue virus infection.

## 1 Data Description

**Fig. 1:**
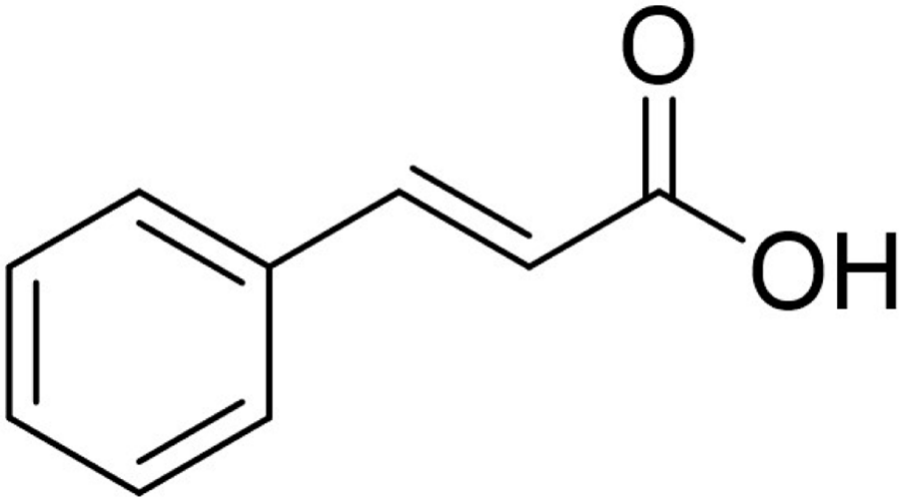
Structure of cinnamic acid.

**Fig. 2:**
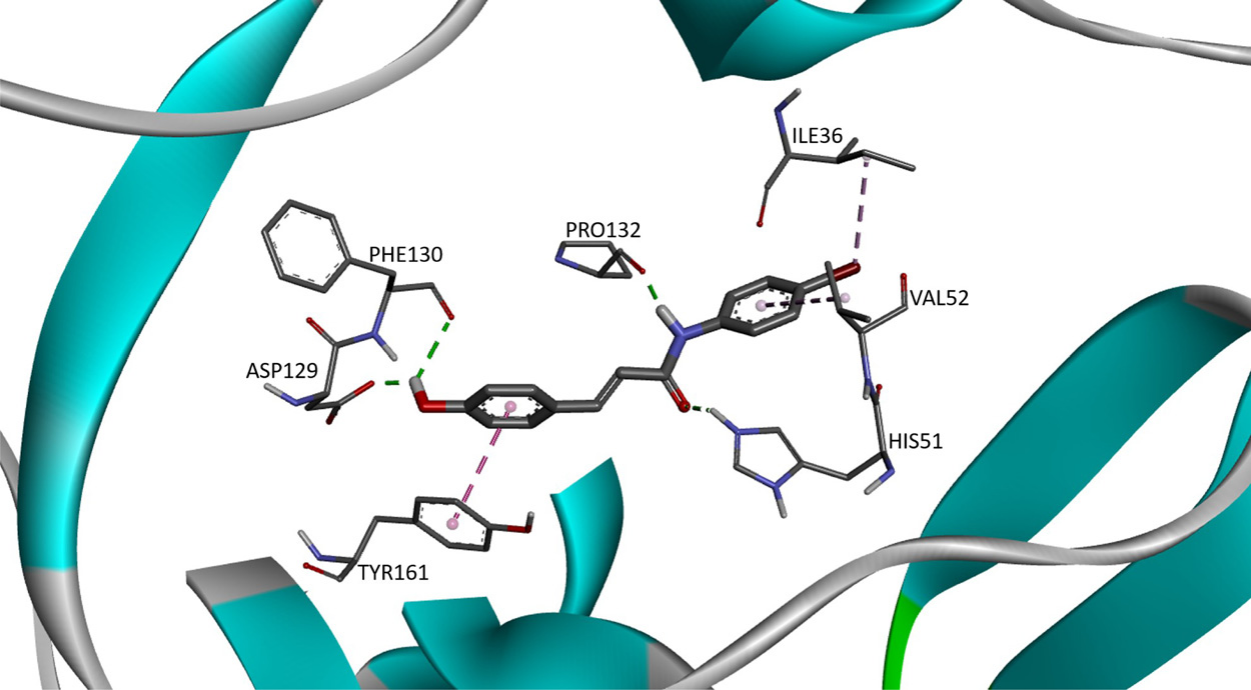
The 3D interaction of compound CAA15 (FEB: −7.22) against viral target NS2B/NS3pro.

**Fig. 3:**
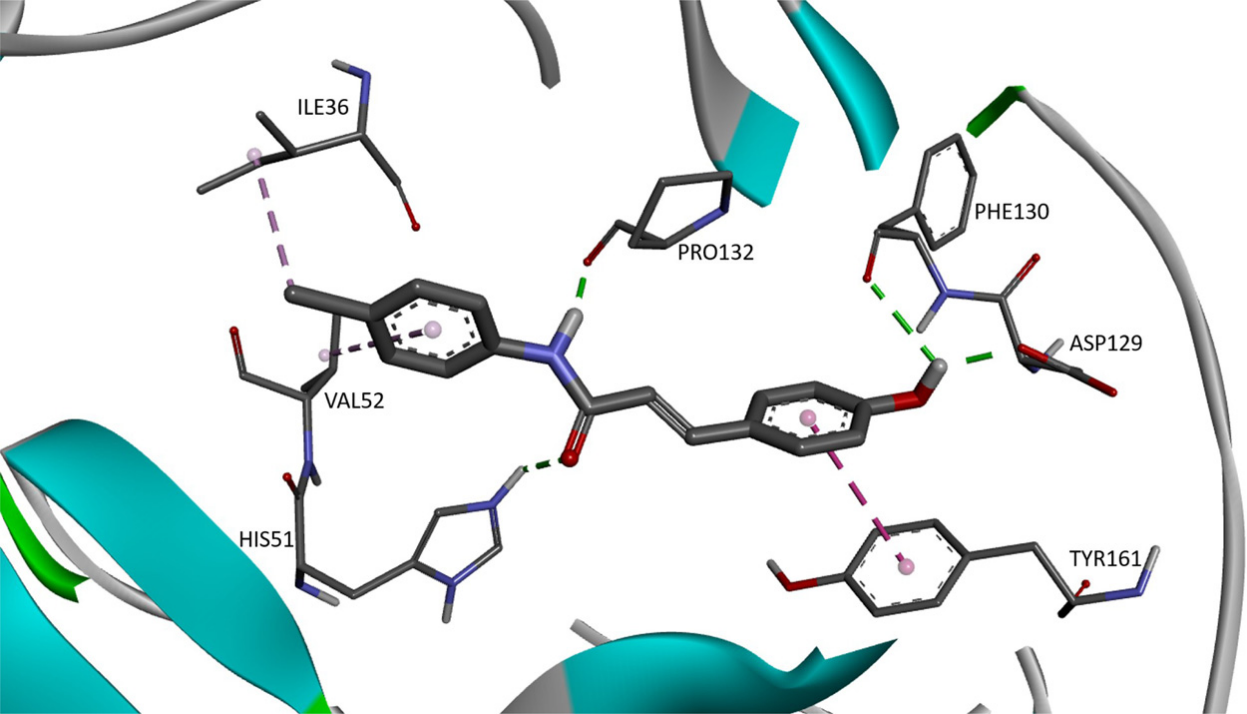
The 3D interaction of compound CAA16 (FEB: −7.03) against viral target NS2B/NS3pro.

**Fig. 4:**
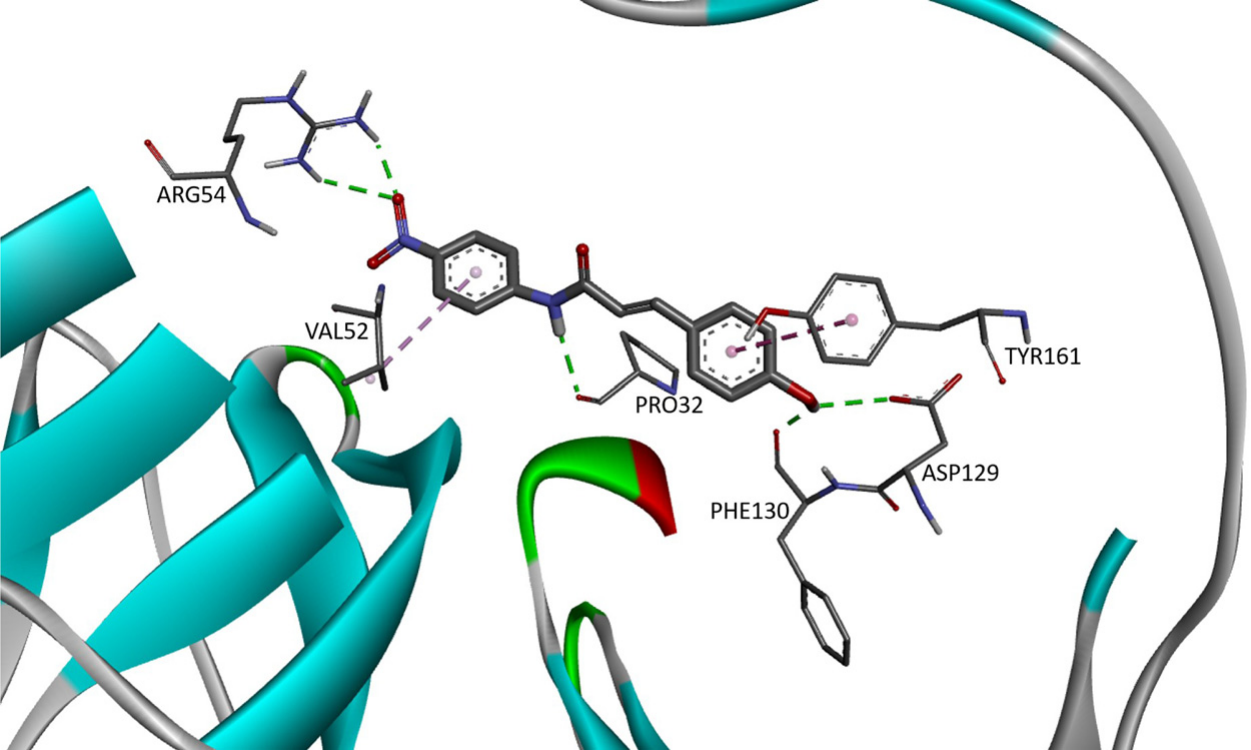
The 3D interaction of compound CAA17 (FEB: −7.07) against viral target NS2B/NS3pro.

The predominant circulating dengue virus (DENV) serotype is dengue virus type 2 (DENV-2). Although many research have been conducted in finding an effective antiviral against it, there is still no specific treatment currently available [1]. Nonetheless, there is emerging interest in development of an effective inhibitor against the NS2b/NS3 serine protease which responsible for seven different polyprotein cleavages in the virus life cycle [2]. Cinnamic acid (Fig. 1) occur in all green plant and is known for their various biological activities [3,4]. The provided docking data of 30 cinnamic amide derivatives against DENV-2 may be useful to develop new drug candidates for the treatment of DENV infections. In this article, Table 1 provides the details of the viral target (as retrieved from Wichapong et al [5].), Table 2 provides the structure of cinnamic amide derivatives, while the free binding energy, interaction type and bond length of the docking are shown in Table 3. The 3D interaction of the top 3 best-docked compounds with the target are shown in Fig. 2, Fig. 3, Fig. 4. The selection was made based on the lowest free binding energy with the highest number of Hydrogen bonding.

**Table 1:** DENV target used in docking.

	Protein	PDB ID	Resolution
1.	NS2B-NS3 Protease	Model – Homologous crystal structure of DENV-2 NS2B/NS3pro [4]	–

**Table 2:** List of cinnamic acids and cinnamic amide derivatives.

No.	Ligand	Structure
1.	CAA01	
2.	CAA02	
3.	CAA03	
4.	CAA04	
5.	CAA05	
6.	CAA06	
7.	CAA07	
8.	CAA08	
9.	CAA09	
10.	CAA10	
11.	CAA11	
12.	CAA12	
13.	CAA13	
14.	CAA14	
15.	CAA15	
16.	CAA16	
17.	CAA17	
18.	CAA18	
19.	CAA19	
20.	CAA20	
21.	CAA21	
22.	CAA22	
23.	CAA23	
24.	CAA24	
25.	CAA25	
26.	CAA26	
27.	CAA27	
28.	CAA28	
29.	CAA29	
30.	CAA30

**Table 3:** Cinnamic amide derivatives and their interactions.

Sr. No.	Ligand	Free Binding energy, FEB (kcal/mol)	Interaction	Type of interaction	Bond distance (Å)
1.	CAA01	−6.66	ILE B:36	π-Alkyl	4.94
				π-Lone pair	2.81
			VAL B:52	π-Alkyl	5.24
			ASP B:129	Carbon H-Bond	3.01
			PHE B:130	H-Bond	2.71
			PRO B:132	H-Bond	2.18
				π-Alkyl	5.19
			TYR B:161	π-π Stacked	5.62
				π-Sigma	3.66

2.	CAA02	−6.56	ILE B:36	π-Alkyl	5.28
				Pi-Lone pair	2.89
			VAL B:52	π-Alkyl	2.35
			HIS B:51	H-Bond	2.08
			SER B:135	H-Bond	1.90
			TYR B:150	π-Alkyl	4.82

3.	CAA03	−6.60	ILE B:36	Alkyl	3.94
				π-Alkyl	5.27
			VAL B:52	π-Alkyl	5.34
			PRO B:132	H-Bond	1.98

4.	CAA04	−6.45	ILE B:36	Alkyl	3.83
				π-Alkyl	5.39
			VAL B:52	π-Alkyl	5.31
			PRO B:132	H-Bond	2.00

5.	CAA05	−6.85	ARG B:54	3 H-Bonds	1.75, 2.37, 2.63
			PRO B:132	H-Bond	2.51
			TYR B:150	π-Alkyl	5.22
			TYR B:161	π-Alkyl	5.17

6.	CAA06	−6.01	HIS B:51	Carbon H-Bond	3.26
				π-π Stacked	5.45
			ASP B:75	Pi-Anion	3.28
			SER A:83	Carbon H-Bond	3.07
			MET A:84	H-Bond	1.95
			ASN B:152	2 H-Bonds	1.78, 2.00
			GLY B:153	Pi-Donor H-Bond	3.07

7.	CAA07	−6.66	TRP B:50	π-Alkyl	5.07
			VAL B:72	Alkyl	4.77
			ILE A:86	Alkyl	4.06
			GLY B:153	H-Bond	2.05
			VAL B:154	π-Sigma	3.72
				Alkyl	4.16
			VAL B:155	H-Bond	1.91
				π-Alkyl	5.07
				Alkyl	5.07

8.	CAA08	−6.81	ILE B:36	π-Alkyl	5.29
				Alkyl	3.61
				Pi-Lone pair	2.95
			HIS B:51	H-Bond	2.04
			VAL B:52	π-Alkyl	5.25
			SER B:135	H-Bond	1.86
			TYR B:150	π-Alkyl	4.81
9.	CAA09	−6.75	ILE B:36	π-Alkyl	5.45
				Alkyl	3.85
			HIS B:51	H-Bond	1.98
			VAL B:52	π-Alkyl	5.31
			PRO B:132	H-Bond	1.85
			TYR B:150	π-Alkyl	5.27
			TYR B:161	π-Sigma	3.74
				π-π Stacked	4.53

10.	CAA10	−6.61	ILE B:36	Π-Alkyl	5.24
				Alkyl	3.55
			PRO B:132	H-Bond	1.83
				π-Alkyl	5.09
			TYR B:161	π-Alkyl	3.61

11.	CAA11	−6.72	HIS B:51	H-Bond	2.11
				π-Cation	4.83
				π-π T-shaped	5.20
			ARG B:54	3 H-Bonds	1.85, 2.56, 2.60
			PRO B:132	π-Alkyl	5.22
			SER B:135	H-Bond	2.20
			TYR B:161	π-π T-shaped	3.90
				π-Sigma	5.11

12.	CAA12	−5.99	ILE B:36	π-Alkyl	5.23
				Alkyl	3.47
			VAL B:52	π-Alkyl	5.24
			PRO B:132	H-Bond	1.98
			GLY B:151	H-Bond	1.98

13.	CAA13	−6.66	ILE B:36	π-Alkyl	5.26
				Alkyl	4.46
				π-Lone pair	2.95
			HIS B:51	H-Bond	2.18
			VAL B:52	π-Alkyl	5.24
			ASP B:29	H-Bond	2.76
			PHE B:130	H-Bond	1.97
			PRO B:132	H-Bond	1.81
			TYR B:161	π-π Stacked	4.67

14.	CAA14	−7.05	HIS B:51	π-Alkyl	3.87
			VAL B:52	π-Alkyl	5.33
				Alkyl	3.58
			ASP B:29	H-Bond	2.39
			PHE B:130	H-Bond	2.07
			PRO B:132	H-Bond	1.97
				Alkyl	4.62
			TYR B:161	π-π Stacked	4.35

15.	CAA15	−7.22	ILE B:36	Alkyl	4.15
			HIS B:51	H-Bond	2.13
			VAL B:52	π-Alkyl	5.42
			ASP B:29	H-Bond	1.91
			PHE B:130	H-Bond	2.43
			PRO B:132	H-Bond	1.73
			TYR B:161	π-π Stacked	4.15
16.	CAA16	−7.03	ILE B:36	Alkyl	4.14
			HIS B:51	H-Bond	2.17
			VAL B:52	π-Alkyl	5.37
			ASP B:29	H-Bond	1.93
			PHE B:130	H-Bond	2.56
			PRO B:132	H-Bond	1.69
			TYR B:161	π-π Stacked	4.16

17.	CAA17	−7.07	VAL B:52	π-Alkyl	5.47
			ARG B:54	2 H-Bonds	2.02, 2.68
			ASP B:29	H-Bond	2.73
			PHE B:130	H-Bond	1.97
			PRO B:132	H-Bond	2.20
			TYR B:161	π-π Stacked	4.62

18.	CAA18	−6.23	GLN B:35	H-Bond	2.34
			ILE B:36	π-Lone pair	2.88
				π-Alkyl	5.20
			VAL B:52	π-Alkyl	5.23
			ASP B:29	H-Bond	2.99
			PHE B:130	H-Bond	1.96
			PRO B:132	H-Bond	1.81
			TYR B:161	π-π Stacked	5.13

19.	CAA19	−7.74	TRP B:50	π-Alkyl	5.04
			VAL B:72	Alkyl	4.83
			ILE A:86	Alkyl	4.03
			GLY B:153	H-Bond	2.05
			VAL B:154	π-Sigma	3.69
				Alkyl	4.09
			VAL B:155	H-Bond	1.90
				π-Alkyl	5.07
				Alkyl	5.12

20.	CAA20	−7.18	TRP B:50	π-Alkyl	5.08
			VAL B:72	Alkyl	4.91
			GLY B:153	H-Bond	2.09
			VAL B:154	π-Alkyl	5.40
			VAL B:155	π-Alkyl	4.83
				Alkyl	5.03
			TYR B:161	π-Alkyl	5.00

21.	CAA21	−7.36	TRP B:50	π-Alkyl	4.97
			HIS B:51	π-π Stacked	4.44
			VAL B:72	Alkyl	4.97
			ASP B:75	π-Cation	3.85
			PHE B:130	Halogen	3.10
			GLY B:153	H-Bond	2.12
			TYR B:161	π-Alkyl	3.98
				π-π Stacked	4.10

22.	CAA22	−7.14	TRP B:50	π-Alkyl	5.10
			HIS B:51	π-π Stacked	4.36
			VAL B:72	Alkyl	5.07
			ASP B:75	π-Anion	3.97
			GLY B:153	H-Bond	2.12
			TYR B:161	π-Alkyl	4.21
				π-π Stacked	4.18
23.	CAA23	−7.24	ILE B:36	π-Alkyl	4.86
			ARG B:54	H-Bond	2.28
			PRO B:132	π-Alkyl	4.61
			SER B:135	Carbon H-Bond	4.36
			TYR B:161	π-Alkyl	4.48
				2 π-Sigma	3.71, 3.90

24.	CAA24	−6.56	SER B:135	3 H-Bonds	1.99, 2.10, 3.01
			VAL B:155	π-Alkyl	4.97

25.	CAA25	−6.70	GLN B:35	Halogen	3.08
			ILE B:36	π-Alkyl	4.95
				Alkyl	4.97
				π-Lone pair	2.80
			VAL B:52	π-Alkyl	5.35
			ASP B:29	Carbon H-Bond	2.97
			PHE B:130	H-Bond	2.76
			PRO B:132	H-Bond	2.12
				π-Alkyl	5.22
			TYR B:161	π-Sigma	3.71
				π-π Stacked	5.58

26.	CAA26	−6.75	GLN B:35	2 Halogen	2.99, 3.68
			ILE B:36	π-Alkyl	5.38
				Alkyl	5.26
				π-Lone pair	2.87
				Carbon H-Bond	4.14
				Halogen	3.37
			VAL B:52	π-Alkyl	5.43
			HIS B:51	H-Bond	2.19
			PRO B:132	Alkyl	5.03
			SER B:135	H-Bond	1.92
			TYR B:150	π-Alkyl	4.75

27.	CAA27	−6.70	ILE B:36	π-Alkyl	5.25
				Alkyl	3.95
			VAL B:52	π-Alkyl	5.32
			ASP B:29	2 Halogens	2.78, 3.46
			PHE B:130	Halogen	3.70
			PRO B:132	H-Bond	1.97
			TYR B:161	π-Alkyl	3.81

28.	CAA28	−6.52	ILE B:36	π-Alkyl	5.26
				Alkyl	3.89
			VAL B:52	π-Alkyl	5.35
			ASP B:29	2 Halogens	2.77, 3.65
			PRO B:132	H-Bond	1.95
			TYR B:161	π-Alkyl	3.70

29.	CAA29	−6.95	ARG B:54	2 H-Bonds	1.79, 2.19
			ASP B:29	2 Halogens	3.03, 3.60
			PHE B:130	2 Halogens	3.15, 3.46
			PRO B:132	H-Bond	2.84
				π-Alkyl	5.31
			TYR B:161	π-Alkyl	3.75
				π-Lone pair	2.89
				π-π Stacked	5.48
30.	CAA30	−5.89	HIS B:51	H-Bond	2.90
			SER A:83	2 Halogens	3.52, 3.60
			MET A:84	H-Bond	2.07
				2 Halogens	2.07, 2.61
			SER B:135	H-Bond	2.76
			ASN B:152	Halogen	3.23
			GLY B:153	Halogen	3.01
			VAL B:155	Alkyl	5.36

## 2 Experimental Design, Materials and Methods

### 2.1 . Selection and retrieval of targets structures

The virtual screening was carried out on the homology model of the dengue virus's non-structural protein, NS2B/NS3pro developed by Wichapong et al [5]. The DENV-2 NS2B/NS3pro model was built based on the DENV-2 complex cofactor-protease using the crystal structure of NS2B/NS3pro West Nile Virus (WNV) as the template. The protein structure was prepared as a macromolecule prior to docking using AutoDock version 1.5.6 package (www.autodock.scrips.edu). Briefly, the protein preparation was done by removing the native ligand, tetrapeptide inhibitor (Bz-Nle-Lys-Ar-H) and water molecules, the addition of polar hydrogen and Kollmann charges.

### 2.2 Ligand preparation and molecular docking

The 3D structures of 30 cinnamic amide derivatives were constructed and energetically optimized using ChemDraw Professional 16.0. The minimised structures were saved in sdf format before being converted into pdb format using OpenBabel-3.1.1 software [6].

The validation of docking protocol was done by re-docking the inhibitor tetrapeptide (Bz-Nle-Lys-Ar-H) with the RMSD value not greater than 2.0 Å. The ligands were prepared by merging of non-polar hydrogen and assigned Gasteiger charged. The center of the grid box was employed around the protease active site at 23.038, 43.372, −0.316 in x, y, and z coordinate, respectively, with a box size of 60 × 60 × 60 dimensions and grid spacing 0.375 Å. The docking of ligands was run with the Lamarckian Genetics Algorithm (GA) search program applied to generate 100 runs. The binding modes of compounds were analyzed using Discovery Studio Client 2020 (www.accelrys.com).

The identification of hit compound was identified based on the conformations with the ones of lowest free binding energy and of the most populated cluster.

## CRediT authorship contribution statement

**Nadia Mohamed Yusoff:** Data curation, Investigation, Methodology, Software, Writing – original draft. **Asnuzilawati Asari:** Conceptualization, Supervision. **Siti Nor Khadijah Addis:** Methodology, Validation, Writing – review & editing.

## Declaration of Competing Interest

The authors declare that they have no known competing financial interests or personal relationships which have or could be perceived to have influenced the work reported in this article.

## Appendix Supplementary materials

Image, application 1
